# HIV and drug abuse mediate astrocyte senescence in a β‐catenin‐dependent manner leading to neuronal toxicity

**DOI:** 10.1111/acel.12593

**Published:** 2017-06-13

**Authors:** Chunjiang Yu, Srinivas D. Narasipura, Maureen H. Richards, Xiu‐Ti Hu, Bryan Yamamoto, Lena Al‐Harthi

**Affiliations:** ^1^ Department of Immunology and Microbiology Rush University Medical Center Chicago IL 60612 USA; ^2^ Department of Pharmacology Rush University Medical Center Chicago IL 60612 USA; ^3^ Department of Pharmacology and Toxicology Indiana University School of Medicine Indianapolis IN 46202 USA

**Keywords:** HIV, methamphetamine, astrocyte, senescence, β‐catenin

## Abstract

Emerging evidence suggests that cell senescence plays an important role in aging‐associated diseases including neurodegenerative diseases. HIV leads to a spectrum of neurologic diseases collectively termed HIV‐associated neurocognitive disorders (HAND). Drug abuse, particularly methamphetamine (meth), is a frequently abused psychostimulant among HIV+ individuals and its abuse exacerbates HAND. The mechanism by which HIV and meth lead to brain cell dysregulation is not entirely clear. In this study, we evaluated the impact of HIV and meth on astrocyte senescence using *in vitro* and several animal models. Astrocytes constitute up to 50% of brain cells and play a pivotal role in marinating brain homeostasis. We show here that HIV and meth induce significant senescence of primary human fetal astrocytes, as evaluated by induction of senescence markers (β‐galactosidase and p16^INK^ ^4A^), senescence‐associated morphologic changes, and cell cycle arrest. HIV‐ and meth‐mediated astrocyte senescence was also demonstrated in three small animal models (humanized mouse model of HIV/NSG‐huPBMCs, HIV‐transgenic rats, and in a meth administration rat model). Senescent astrocytes in turn mediated neuronal toxicity. Further, we show that β‐catenin, a pro‐survival/proliferation transcriptional co‐activator, is downregulated by HIV and meth in human astrocytes and this downregulation promotes astrocyte senescence while induction of β‐catenin blocks HIV‐ and meth‐mediated astrocyte senescence. These studies, for the first time, demonstrate that HIV and meth induce astrocyte senescence and implicate the β‐catenin pathway as potential therapeutic target to overcome astrocyte senescence.

## Introduction

With the introduction of combined antiretroviral therapy (cART), HIV has been transformed from a deadly virus to a chronic infection linked to a number of comorbid conditions associated with an aging population. In particular, HIV‐infected individuals are at an increased risk of neurocognitive and motor impairments (Saylor *et al*., 2016) termed HIV‐associated neurocognitive disorders (HAND). Incidences of HAND are expected to increase as the HIV+ population is living longer and aging. HAND is driven by complex interactions of HIV invasion into the central nervous system (CNS), inflammatory responses in the CNS that ensue, and comorbid factors such as illicit drug abuse. Methamphetamine (meth), in particular, is a potent psychostimulant (Hser *et al*., 2008) and is frequently abused in the HIV/AIDS population. HAND is more severe among HIV+ individuals who are meth abusers than those who are not (Nath *et al*., 2002; Chana *et al*., 2006; Purohit *et al*., 2011). Further, meth users who are HIV negative also experience neurocognitive deficits that persist even following periods of abstinence from meth use (Scott *et al*., 2007; Iudicello *et al*., 2010). The underlying mechanisms by which HIV and meth dysregulate resident brain cells are not entirely clear. We evaluated here the impact of HIV and meth on cellular aging in astrocytes.

Astrocytes play a pivotal role in brain homeostasis. They are the major source of storage for glucose and lactate for energy metabolism in neurons. They are also responsible for > 90% of the uptake of the extracellular neurotransmitter glutamate, which in excess is neurotoxic. Astrocytes release a number of neurotrophic factors, regulate the integrity of the blood–brain barrier, and contribute to immunity within the CNS. Thus, dysfunctions of astrocytes will likely lead to disruption in several brain functions. Further, there is an emerging recognition that astrocytes play a significant role in neurodegenerative diseases. In many of these neurodegenerative diseases, including HAND, reactive/activated astrocyte is a hallmark feature of the disease. Both HIV encephalitis brains and brains of meth abusers have increased levels of astrogliosis (Langford *et al*., 2003). Reactive astrogliosis is also evident in the striatum of a meth‐treated rat model (Pu & Vorhees, 1993). While a certain degree of astrocytosis is neuroprotective, overly reactive astrocytes compromise their protective properties such as alteration in ion transport (Benos *et al*., 1994) and impaired glutamate transport (Wang *et al*., 2003), which lead to harmful neuroinflammatory processes such as increased levels of IL‐1β, IL‐6, and TNF‐α (Minagar *et al*., 2002; Shah *et al*., 2012).

Senescence is a cellular aging and stress response. The senescent cells are characterized by their flat morphology in culture and expression of senescence‐associated β‐galactosidase (SA‐βGal) activity and p16^INK4A^ (Campisi, 2013). p16^INK4A^, also known as cyclin‐dependent kinase inhibitor 2A/multiple tumor suppressor 1, is an inhibitor of cyclin D‐dependent kinases such as CDK4 and CDK6. Activation of CDK4 and CDK6 phosphorylates pRB and release of E2F thus promotes cell cycle progression. Senescence may exhaust progenitor/stem cells and promote inflammatory responses. Cellular senescence may also exacerbate age‐related phenotypes *in vivo*, as elimination of senescent cells in a conditioned knockout mouse model effectively delays aging‐associated disorders (Baker *et al*., 2011). Human astrocytes can undergo senescence in culture, exhibiting classical phenotypes of senescent cells such as enhanced expression of SA‐βGal, p16^INK4A^, and having a flat morphology (Bhat *et al*., 2012). Astrocyte senescence may contribute to brain aging (Kang *et al*., 2015) and neurodegenerative diseases such as Alzheimer's (Bhat *et al*., 2012) and Parkinson's disease (Chinta *et al*., 2013). Although HIV induces cellular senescence of immune T cells, contributing to dysfunction of T cells (Deeks *et al*., 2012), it is unclear whether senescence plays a role in the dysfunction of HIV‐infected brain. Similarly, meth affects gene expression involved in cell cycle in human primary astrocytes (Jackson *et al*., 2014); however, little is known about the potential impact of HIV and meth on astrocyte senescence.

β‐Catenin is a central mediator of the Wnt/β‐catenin pathway. The Wnt/β‐catenin signaling pathway is vital to various functions in the CNS including neurogenesis, neurotransmitter release, induction of long‐term potentiation and depolarization resulting in increased synaptic strengths, and memory consolidation (Al‐Harthi, 2012). Dysregulation of Wnt/β‐catenin signaling is linked to a number of neurodegenerative diseases, including Alzheimer's and Parkinson's disease, and psychiatric disorders such as bipolar disorder and depression (Al‐Harthi, 2012; Berwick & Harvey, 2012; Inestrosa *et al*., 2012; Levchenko *et al*., 2015). Astrocytes secrete robust levels of Wnt ligands (Richards *et al*., 2015), which are glycoproteins that bind to Frizzled receptors and the coreceptor low‐density lipoprotein receptor‐related protein (LRP) 5/6 leading to destabilization of the β‐catenin destruction complex. Hypophosphorylated β‐catenin translocates to the nucleus, where it interacts with TCF/LEF to displace its corepressors and recruit either positive or negative transcription cofactors to regulate Wnt target genes. We previously demonstrated that endogenous Wnt/β‐catenin signaling in astrocytes is a restriction pathway to productive HIV replication. Suppression of β‐catenin relieves this transcriptional restriction to HIV and promotes higher level of HIV replication in astrocytes. Further, HIV comorbid factor meth suppresses β‐catenin signaling in human primary fetal astrocytes (HFAs; Sharma *et al*., 2011), which potentially could increase astrocyte senescence. β‐Catenin regulates cyclin D expression in human astrocytes (Narasipura *et al*., 2012). Cyclin D‐dependent CDK4 and CDK6 play a critical role in cellular senescence. These collective observations suggest that β‐catenin may be an important determinant mediating HIV‐ and meth‐induced cell senescence in astrocytes.

## Results

### Meth and HIV induce senescence in primary human fetal astrocytes (HFAs)

**Figure 1:**
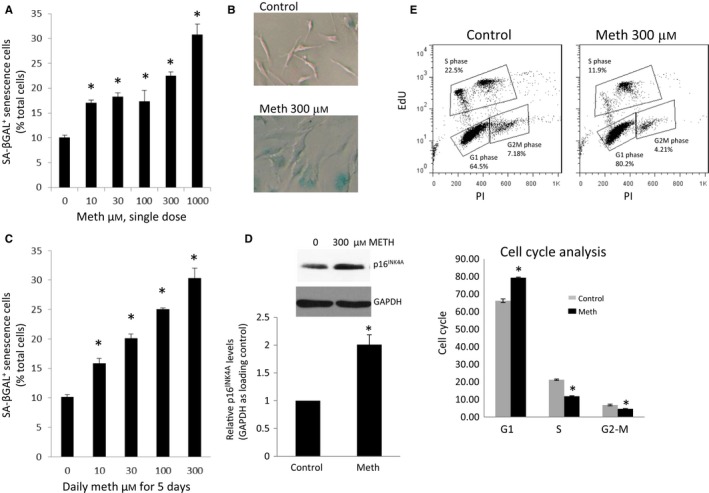
Meth increases senescence in HFAs. (A) HFAs were treated with meth at different concentrations, 0, 10, 30, 100, 300, and 1000 μm. At day 6, cells stained for SA‐βGal were imaged, and SA‐βGal‐positive cells were quantitated using ImageJ software as percentage of total cells. * denotes *P* < 0.05. *n* = 4. (B) HFAs were treated with 300 μm meth. A representative image of SA‐βGal‐positive cells is shown in blue at day 6. (C) HFAs were treated daily with meth at different concentrations, 0, 10, 30, 100, 300, and 1000 μm. At day 6, cells were stained for SA‐βGal. Data analysis was performed as in A. (D) HFAs were treated as in C. At day 6, cell lysates were analyzed for the levels of p16^INK^ ^4A^ with Western blotting, and normalized to GAPDH. The value of the control cells set for 100%. * denotes *P* < 0.05. *n* = 3. E) HFAs were treated daily with 300 μm of meth. At day 6, cell cycle analysis was performed using Click‐iT EdU by Flow Cytometry. G1 denotes G1 phase in the cell cycle, S for synthesis phase, and G2/M for G2 and mitotic phases. * denotes *P* < 0.05. *n* = 4.

We first examined whether meth induces senescence of HFAs. human fetal astrocytes were treated with a single dose of meth (0–1000 μm); at day 6, HFAs were stained for SA‐βGal. Meth significantly induced SA‐βGal expression even at the lower meth dose of 10 μm (Fig. 1A). The greatest induction of SA‐βGal was observed at 1000 μm meth, which while excessive, during binges episodes, meth concentration could reach 165–776 μm in the brain (Talloczy *et al*., 2008). Further, meth‐treated HFAs demonstrated a classical flat morphology of senescent astrocytes (Fig. 1B). Given that meth abusers commonly binge on meth in consecutive days, we exposed HFAs to daily meth doses (0–300 μm) for 5 days, and at day 6 after first meth treatment, SA‐βGal expression was measured. At lower doses of daily meth treatments (10–30 μm), meth did not enhance SA‐βGal expression above what was observed with equivalent single‐dose treatment (Fig. 1C). However, at higher meth doses of daily treatment (100–300 μm), meth enhanced SA‐βGal expression in comparison with the single‐dose treatment, as a daily 100 μm meth induced Sa‐βGal at 8% higher than 100 μm single meth dose (17% vs. 25%), a daily 300 μm meth 9% higher than 300 μm single meth dose (31% vs. 22%). Meth also increased the expression of another marker of cell senescence, p16^1NK4A^, as evaluated by Western blot (Fig. 1D). As senescent cells do not proliferate, we evaluated the impact of meth on the cell cycle. HFAs were treated with a daily dose of meth at 300 μm and at day 5 stained with EdU and PI. Meth reduced S phase cells by twofold (11% vs. 21%) in comparison with vehicle‐treated cells, while induced cells in G1 phase by 16% (81% vs. 65%; Fig**.** 1E). Together, these data demonstrate that meth induces cell senescence as demonstrated by induction in biomarkers for cell senescence (SA‐βGal and p16^INK4A^) and cell cycle arrest.

**Figure 2:**
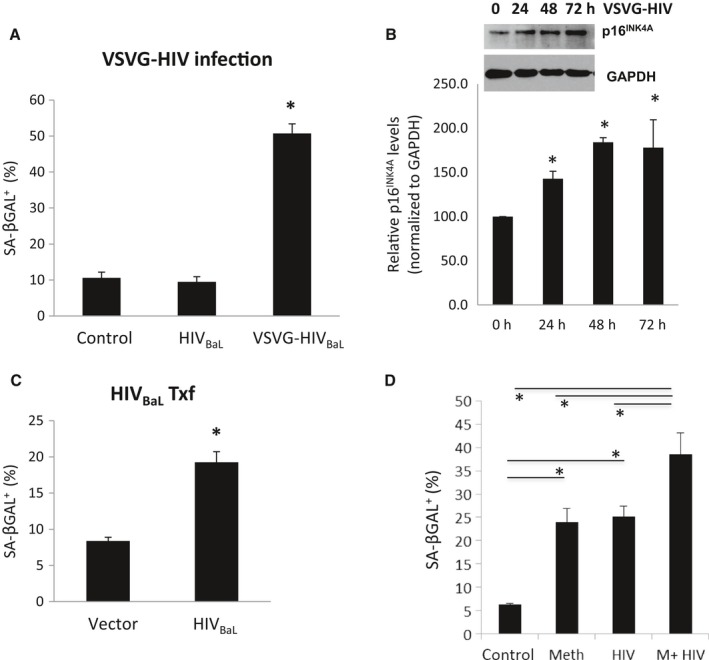
HIV induced HFA senescence *in vitro*. HIV‐1 strain (HIV_B__aL_) was pseudotyped with vesicular stomatitis virus envelop protein G (VSVG). HFAs were infected with VSVG‐pseudotyped HIV_B__aL_ (VSVG‐HIV) at 20 ng per million cells or mock infected. At day 6, cells were fixed and stained for SA‐βGal (A) or Western blot performed for p16^INK^ ^4A^ and normalized to GAPDH (B). HFAs were transfected with HIV_B__al_ wild‐type expression plasmid, or a control plasmid pcDNA3^+^ (C). At day 6 post‐transfection, the cells were stained for SA‐βGal. D) HIV and meth additively increased HFA senescence. HFAs treated with control 1xPBS, 300 μm of meth, VSVG‐HIV at 10 ng per million cells, or 300 μm meth + VSVG‐HIV at 10 ng per million cells. Next day, cells were extensively washed and then treated daily with 1xPBS, 300 μm meth, 1xPBS, or 300 μm meth. At day 6, cells were stained for SA‐βGal. * denotes *P* < 0.05. *n* = 3–4.

To assess the impact of HIV on astrocyte senescence, HFAs were either infected with a VSVG‐pseudotyped HIV_BaL_ (VSVG‐HIV, 20 ng p24 per million cells) or mock infected. VSVG‐HIV induced SA‐βGal in HFAs by fivefold (50% vs. 10%) in comparison with VSVG alone (Fig. 2A). Infection of VSVG‐HIV also caused a 1.8‐fold increase in p16^INK4A^ levels (Fig. 2B). HFAs were also transfected with HIV_BaL_ expression plasmid. Transfection of HFAs with HIV_Bal_ plasmid induced SA‐βGal^+^ cells from 8% in pcDNA3 control plasmid to 20% in HIV‐transfected cells (Fig. 2C). To determine whether meth would increase senescence in HIV‐infected HFAs, HFAs were infected with VSVG‐HIV (10 ng p24 per million cells) and treated with a daily dose of meth at 300 μm for 5 days. While meth alone and HIV alone induced SA‐βGal expression by approximately 22% in comparison with untreated cultures, together, they had an additive effect on SA‐βGal induction (Fig. 2D).

### HIV and meth inhibit β‐catenin signaling pathway

To assess the mechanism(s) that may be driving astrocyte senescence in HIV infection and meth treatments of astrocytes, we evaluated the role of β‐catenin in these responses. We previously demonstrated that β‐catenin signaling is robustly expressed in astrocytes (Richards *et al*., 2015) and, albeit in progenitor‐derived human astrocytes, that meth and HIV Tat suppress β‐catenin signaling (Sharma *et al*., 2011; Henderson *et al*., 2012). Wnt signaling pathway regulates senescence, as repression of Wnt2 induced senescence in stressed primary human fibroblast WI38 cells (Ye *et al*., 2007) and knock‐down of SFRP1 inhibited stress‐induced cell senescence (Elzi *et al*., 2012). We therefore evaluated whether meth‐ and HIV‐mediated downregulation of β‐catenin signaling induces cell senescence in HFAs.

**Figure 3:**
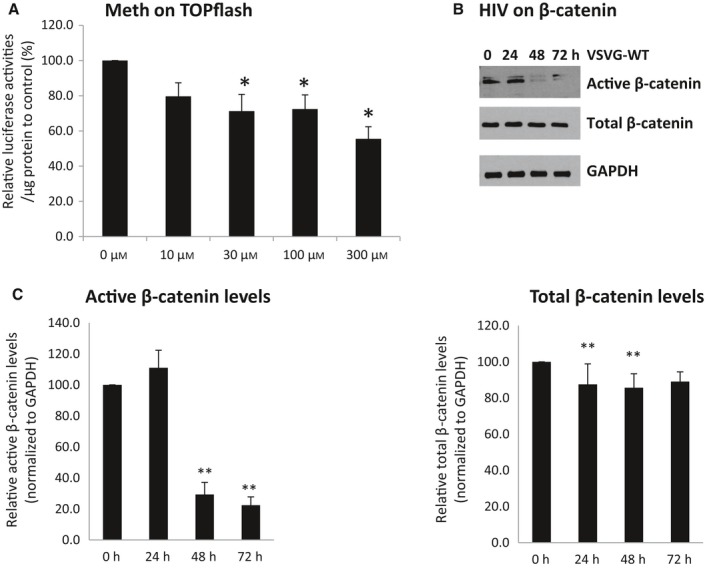
Meth and HIV infection decrease β‐catenin signaling. A) HFAs were transfected with TOPflash and treated daily with 300 μm of meth. At 48 h post‐transfection, luciferase activities were measured. The value of arbitrary units per μg of cellular protein in the control was set at 100%. B‐C) HFAs were infected with VSVG‐HIV. Cell lysates were collected postinfection at 0, 24, 48, and 72 h. Western analysis was used for the detection of total β‐catenin and active β‐catenin, normalized to GAPDH. * denotes *P* < 0.05. *n* = 3–5.

Human fetal astrocytes (HFAs) were transiently transfected with a Wnt/β‐catenin signaling reporter plasmid, TOPflash. TOPflash consists of several TCF/LEF DNA binding sites linked to firefly luciferase. TCF/LEF and β‐catenin bind to TCF/LEF DNA sequences to regulate gene expression. Meth at 300 μm inhibited TOPflash activities by approximately 45% (Fig. 3A). These data are consistent with meth inhibition of β‐catenin signaling in progenitor‐derived astrocytes (PDAs) (Sharma *et al*., 2011). HIV also inhibited β‐catenin signaling in HFA, as demonstrated by inhibition of active β‐catenin expression post‐VSVG‐HIV infection to 30% at 48 h and 22% at 72 h of the VSVG control (Fig. 3B‐C). These data demonstrate that meth and HIV (not just HIV Tat) significantly downregulate β‐catenin signaling in HFAs.

### β‐catenin rescues human fetal astrocyte (HFA) from meth‐ and HIV‐induced senescence

**Figure 4:**
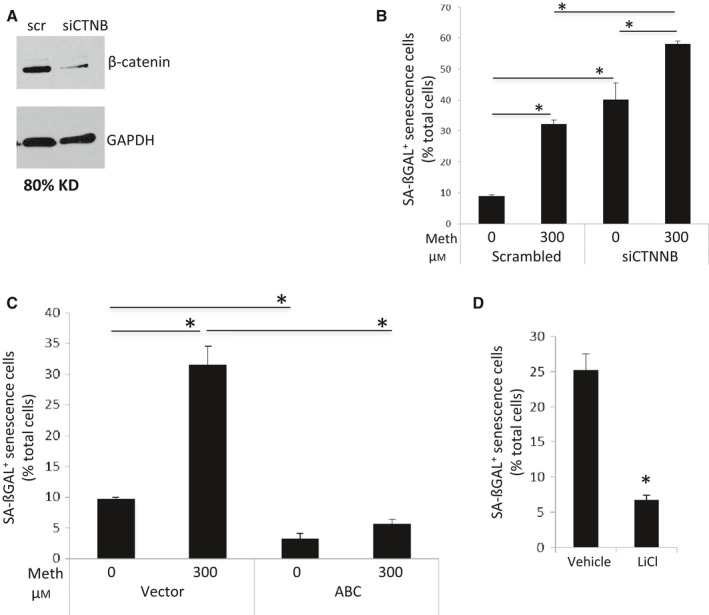
Suppression of β‐catenin induces HFA senescence (A, B). A) HFAs were transfected with scrambled control siRNA or β‐catenin siRNA. At day 3, cell lysates were harvested for analysis of β‐catenin levels by Western blotting, using GAPDH as control. B) In parallel experiments, siRNA‐transfected HFAs were treated with 1xPBS vehicle or 300 μm of meth daily. At day 6, cells were fixed and stained for SA‐βGal. * denotes *P* < 0.05. *n* = 4. In contrast, increased β‐catenin levels rescued HFAs from senescence induced by meth and HIV (C, D). C). HFAs were transfected with control plasmid pcDNA3 + , or a plasmid expressing constitutive active β‐catenin (S35Y). Transfected HFAs were treated with 1xPBS vehicle or 300 μm of meth daily. At day 6, cells were stained for SA‐βGal. D) HFAs were either infected with VSVG‐HIV or mock infected. Next day, cells were washed extensively and cultured in cABM medium in the presence of 5 mm LiCl or control vehicle 1xPBS for 6 days. HFAs were then analyzed for SA‐βGal expression. * denotes *P* < 0.05. *n* = 4.

To directly address whether downregulation of β‐catenin induces cell senescence in HFAs, HFAs were transfected with β‐catenin siRNA or a scrambled siRNA (Narasipura *et al*., 2012). Efficiency of β‐catenin knockdown is typically greater than 80% (Fig. 4A). Knockdown of β‐catenin induced SA‐βGal expression by fourfold (40% vs. 9%) (Fig. 4B) while knockdown of β‐catenin together with meth increased SA‐βGal expression to 58%. To assess whether inducing β‐catenin would block/protect HFAs from either meth‐ or HIV‐mediated cell senescence, HFAs were transfected with a constitutive active β‐catenin plasmid or control plasmid. This plasmid, S35Y β‐catenin, has a serine at 35 to tyrosine mutation preventing β‐catenin phosphorylation by destruction complex and subsequent proteasomal degradation. Induction of β‐catenin blocked meth‐mediated HFA senescence by fivefold (Fig. 4C). Interestingly, it also reduced basal levels of SA‐βGal expression by threefold (3% vs. 10%) without meth treatment. To induce β‐catenin in HIV‐infected HFA, cells were treated with LiCl (5 mm), which induces β‐catenin expression by inhibiting GSK3β, a component of the β‐catenin destruction complex. Treatment of HIV‐infected HFAs with LiCl rescued SA‐βGal expression to baseline (approximately 6%, Fig. 4D). These data demonstrate that induction of β‐catenin rescues HFA from signals that induce senescence in this case of either meth or HIV infection.

### Senescent human fetal astrocytes (HFAs) induce neurotoxicity

**Figure 5:**
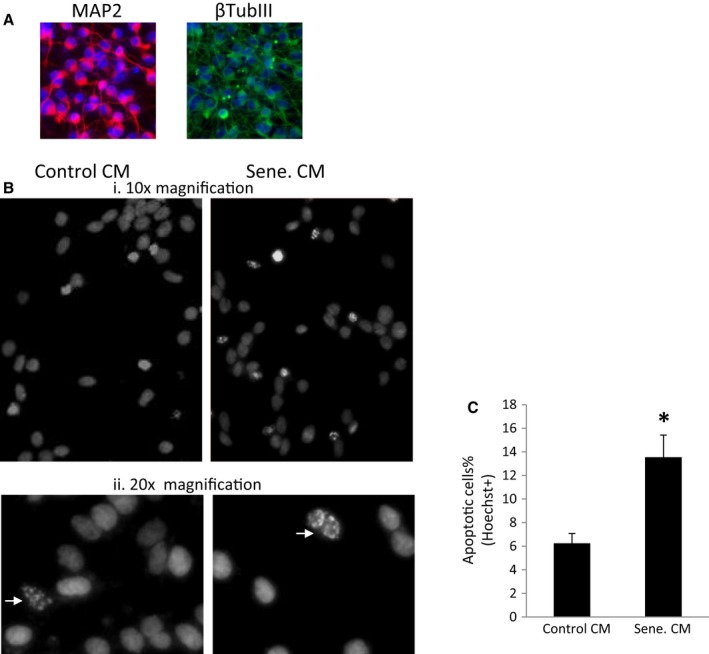
Conditioned medium of senescent HFAs induces neurotoxicity. (A). differentiated LUHMES cells at day 5 were stained for MAP2 and the neurofilament tubulin III protein. B, HFAs were treated with the control vehicle or meth to induce senescence. LUHMES cells were differentiated into human dopaminergic neurons for 2 days. At day 3, media were changed to the HFA conditioned media. At day 5, neurons were stained with Hoechst dye. Apoptotic neurons were detected as fragmented nuclei staining, indicated by arrows. The apoptotic neurons were imaged and quantitated (C). * denotes *P* < 0.05. *n* = 4.

Senescent cells exhibit an altered profile of secreted cytokines and chemokines, a phenomenon termed the senescence‐associated secretion phenotype (SASP), leading to inflammation and neurotoxicity. To explore the consequence of astrocyte senescence on neurons, conditioned media (CM) from senescent astrocytes were added to neurons. Specifically, human LUHMES cells were differentiated into dopaminergic neurons *in vitro* (Scholz *et al*., 2011) and HFAs were treated with meth or vehicle (1xPBS) to induce senescence. The CM from HFAs was then added to LUHMES neurons for 3 days. In parallel, a dose of 300 μm or 600 μm of meth was added directly to neurons cultures with fresh HFA growth medium. Cells were stained with Hoechst dye to detect apoptotic cell death. Direct addition of meth, at these doses, to neurons did not induce cell death, a result consistent with previous report (Scholz *et al*., 2011). However, HFA CM induced neuronal apoptosis by 25% (Fig. 5 A and B), illustrating that astrocyte senescence induces neuronal toxicity.

### Astrocyte senescence *in vivo*

**Figure 6:**
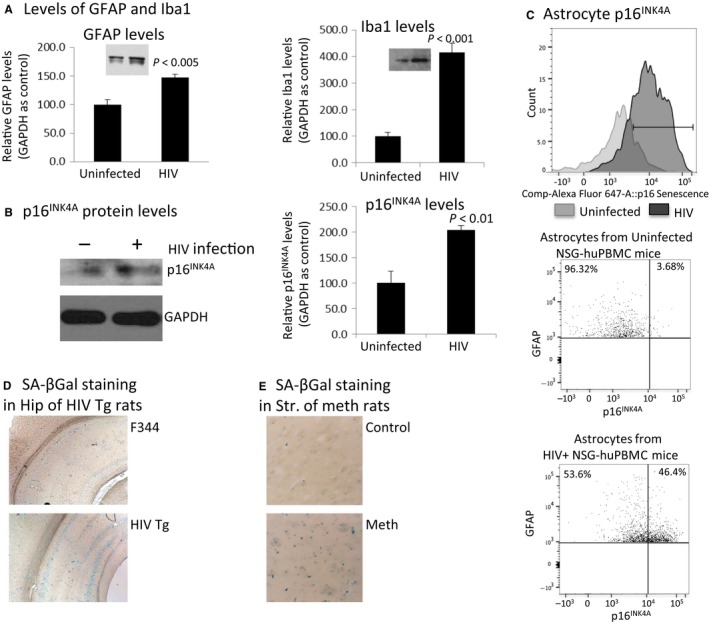
*In v*ivo detection of astrocyte senescence mediated by HIV or meth in three small animal models. A). Brains from HIV+ NSG‐huPBMC mice (*n* = 8) were dissected and brain lysates were extracted with 1xRIPA buffer and analyzed for the levels of GFAP (A) and Iba1 (B), and p16^INK^ ^4A^ (C) by Western blotting. In (D), astrocytes were isolated by Percoll gradient after enzymatic digestion of brain tissue. The astrocytes were immunostained with anti‐GFAP and anti‐p16^INK^ ^4A^ and further labeled with appropriate secondary antibodies conjugated with Alexa‐594 and Alexa‐488, respectively. Astrocytes were then analyzed by flow cytometry. E, brains (*n* = 8) were harvested from adult male HIV‐Tg and non‐Tg rats and stained for SA‐βGal. Images were representative staining in the cortex. F) Adult Sprague Dawley rats were treated with a meth regimen (10 mg/kb, i.p., every 2 h × 4). At day 7, brains were fixed and stained for SA‐βGal. Images were representative staining of SA‐βGal in the striatum.

To determine whether astrocyte senescence occurs *in vivo*, we used IL‐2rγc‐/‐ (NSG) mice reconstituted with human PBLs (huPBLs) and infected with HIV_BaL_ by i.p, as described (Richards *et al*., 2016). Three weeks postinfection, the mice were sacrificed and the brains were analyzed for astrocyte senescence. HIV‐infected NGF‐huPBL mice exhibit enhanced expression of GFAP and Iba1 (Fig. 6A) by 1.5‐ and fourfold, respectively, in the hippocampus of HIV‐infected mice in comparison with mock‐infected mice. Further, p16^INK4A^ protein expression was elevated by twofold in the hippocampus of HIV‐infected mice in comparison with the uninfected control brains (Fig. 6B). To specifically assess elevation of p16^INK4A^ in astrocytes of HIV‐infected humanized mice, astrocytes were isolated from mouse cortex and stained for GFAP and p16^INK4A^ and analyzed by flow cytometry. We demonstrated a twofold induction in p16^INK4A^ expression in astrocytes of HIV‐infected mice in comparison with mock‐infected mice (Fig. 6C). In another HIV small animal model, HIV‐1 transgenic rat (HIV‐Tg) (Reid *et al*., 2001), SA‐βGal expression is robust in the hippocampus of HIV‐Tg at 6 weeks of age, in comparison with non‐Tg control (Fig. 6D).

To examine whether meth induces cell senescence *in vivo*, we utilized a rat model that shows similar neuron damage seen in human meth users, where the animal receives meth at 10 mg mL^−1^, every 2 h for four times (Halpin & Yamamoto, 2012). The striatum is most vulnerable to meth‐induced toxicity. We evaluated SA‐βGal expression in striatum and hippocampus. We observed that in the striatum, SA‐βGal expression was enhanced in meth‐treated animals in comparison with the vehicle control (Fig. 6E), whereas no difference was detected in the hippocampus of meth‐treated rats in comparison with vehicle‐treated rats (data not shown). Together, these data provide *in vivo* evidence using three animal models to show that HIV and meth induce cell senescence in brain.

## Discussion

Cell senescence and its associated SASP may drive premature organism aging (Kennedy *et al*., 2014). Astrocyte senescence is implicated in human brain aging (Kang *et al*., 2015) and in neurodegenerative diseases such as Alzheimer's (Bhat *et al*., 2012) and Parkinson's disease (Chinta *et al*., 2013). HAND exhibits many features of neuroinflammation, which is a common thread among a number of other premature aging and neurodegenerative diseases, yet whether HIV neuroinvasion impacts astrocyte senescence is not clear. We evaluated here the impact of HIV and meth on astrocyte senescence. We show that both HIV and meth induce astrocyte senescence as evaluated by enhanced expression of senescence biomarkers such as SA‐βGAL, p16^INK4A^, predominance of cells in S phase of the cell cycle, and a flat morphology of astrocytes. The HIV and meth effect on astrocyte senescence is additive in nature. Most significantly, when astrocytes were induced into senescence by meth, the conditioned medium increased neuronal apoptosis, which is consistent with neuron damage in chronic meth abusers. Presumably, the conditioned media of senescent astrocytes may either reflect a senescence‐associated secretion phenotype (SASP), which is characterized by elevated levels of potent inflammatory cytokines (Campisi, 2013), or lack key neurotrophic factors relevant to maintaining neuronal health. SASP could also propagate senescence in a paracrine manner. In oncogene‐induced senescence models, both *in vitro* and *in vivo*, increased levels of IL‐1α induced SASP and senescence in neighboring cells (Acosta *et al*., 2013). Thus, it is conceivable that meth‐ and HIV‐induced senescence in astrocytes would amplify senescence in neighboring astrocytes and even neuronal stem cells in the surrounding regions.

Astrocytes are restricted to HIV infection *in vitro*. This restriction can be overcome by priming astrocytes with IFN‐γ, which inhibits β‐catenin signaling in astrocytes, facilitating higher level of HIV replication (Henderson *et al*., 2012). In studying the role of HIV in astrocyte senescence, we were unable to prime astrocytes with IFN‐γ because IFN‐γ alone led to astrocyte senescence (data not shown), suggesting that inflammatory cytokines, which are elevated in HAND, could also lead to enhanced astrocyte senescence. Alternatively, we infected HFA with VSVG‐pseudotyped HIV or transfected the cells with HIV plasmid. Both strategies demonstrated that HIV causes astrocyte senescence *in vitro*. This is not an *in vitro* artifact, as astrocytes from HIV‐infected humanized mice also demonstrated astrocyte senescent phenotype through increased expression of p16^INK4A^. Further, SA‐βGal expression is elevated in the brains of HIV‐Tg rats and in meth‐treated rat brains. Thus, our *in vitro* and *in vivo* data support the concept that HIV and meth mediate astrocyte senescence.

To assess approaches to protect/rescue astrocytes from senescence mediated by meth or HIV, we focused on the Wnt/β‐catenin signaling pathway. Astrocytes have a robust endogenous expression of Wnt/β‐catenin signaling, which is a pro‐survival/pro‐proliferative pathway (Henderson *et al*., 2012). Wnt signaling pathway regulates cell senescence. A switch from β‐catenin‐dependent to β‐catenin‐independent pathway in old mice resulted in senescence of hematopoietic stem cells (Florian *et al*., 2013). Repression of Wnt2 expression triggers early onset of senescence (Ye *et al*., 2007), while knockdown of Wnt signaling inhibitor SFRP1 blocked stress‐induced senescence (Elzi *et al*., 2012). Using gain‐ and loss‐of‐function studies, we demonstrated that β‐catenin protects astrocytes from HIV/meth‐mediated cell senescence. We used LiCl as an inducer of β‐catenin signaling in HIV‐infected astrocytes, instead of transfecting HIV‐infected astrocytes with the constitutively active β‐catenin construct, because infected HFAs were fragile to transfection attempts. Nonetheless, knocking down β‐catenin in uninfected astrocytes induced senescence and activation of β‐catenin by LiCl treatment of HIV‐infected cells rescued them from cell senescence.

β‐catenin activities are central to the cell cycle, regulating multiple proteins essential in the cell cycling such as c‐Myc and Cyclin D in human astrocytes (Narasipura *et al*., 2012). β‐catenin also regulates the expression of p16^INK4A^ in a cell context‐dependent manner (Delmas *et al*., 2007; Bishop *et al*., 2010). During the cell cycle, the active cyclin‐dependent kinases CDK4 and CDK6 complexed with cyclin D phosphorylate RB protein. RB phosphorylation releases E2F transcription factors which activate the expression of genes promoting G1 to S phase entry. Inactivation of β‐catenin would decrease levels of cyclin D and blocks the cell entry to S phase. By directly binding to CDK4 and CDK6, p16^INK4a^ blocks the assembly of CDK4/6 with cyclin D and their activation, resulting in G1 phase arrest. p16^INK4A^ is a critical regulator in the induction of senescence. Thus, Wnt/β‐catenin signaling is central in cell senescence induced by cell stress such as HIV infection and/or meth treatment.

Much attention is focused on direct neuronal injury in HIV and meth comorbidity. Meth causes dopaminergic neuronal toxicity and HIV, depending on stages of disease, can lead to neuronal toxicity or neuronal injury manifested by neuronal pruning and synpatodendritic dysregulation. Our study points to dysregulation of astrocytes, specifically induction of astrocyte senescence in promoting neuronal injury. A greater attention is needed to understand the role of astrocyte senescence in disruption of astrocyte–neuronal communication as a mechanism driving HIV/meth heightened neuropathogenesis and as a common feature in neurodegenerative diseases.

## Experimental Procedures

### Ethical statement of animal research

NOD/SCID/IL‐2rcγ‐/‐ mice were purchased from The Jackson Laboratories (Bar Harbor, ME; stock number 005557); adult male HIV‐1 transgenic Fischer F344 rats (HIV‐1 Tg) and control Fischer F344 rats (non‐Tg) at 3–4 weeks of age from Harlan Laboratory (Indianapolis, IN), and adult male Sprague Dawley rats (180e275 g) from Harlan. The animals were housed under pathogen‐free conditions in accordance with the Institutional Animal Care and Use Committee (IACUC #14‐014, #13‐048, and #106095) at Rush University Medical Center and University of Toledo, and the ethical guidelines for care of laboratory animals at the National Institutes of Health.

### Reagents

Methamphetamine (M8750), LiCl, BIO, and propidium iodide were purchased from Sigma (St. Louis, MO). Access to meth was approved by state and federal regulations. Senescence‐associated β‐galactosidase (SA‐βGal) kit and anti‐GFAP G5 antibody were purchased from Cell Signaling Inc (Danvers, MA). Other antibodies include Iba1 antibody (Fisher Scientific #019‐19741, Pittsburgh, PA), active β‐catenin antibody (US Biological C2069‐47, Salem, MA), total β‐catenin antibody (C2206; Sigma), p16^INK4A^ antibody (Proteintech #22515‐1‐AP, Chicago, IL), GAPDH antibody (G99445; Sigma), antineurofilament heavy chain (Millipore Ab5539, Danvers, MA). Click‐iT Plus EdU Alexa Fluor Flow Cytometry Assay kit (C10646) was from Invitrogen (Waltham, MA).

### Human fetal astrocytes (HFA) propagation and meth treatment

HFAs (~18‐week gestation, Lonza Inc, Walkersville, MD) were cultured in complete astrocyte medium according to the supplier's protocol. Early passages (p2‐p4) of HFAs were used for all experiments. For meth treatment of HFAs, the cells were seeded in 8‐well cell culture slide chambers (Fisher Scientific, Rockford, IL). Media were changed the next day, and the cells were treated with meth (0, 10, 30, 100, 300, 1000 μm) or vehicle control (1xDPBS). Meth was added once or daily at specified dose for 5 days, as indicated.

### Human fetal astrocytes (HFA) infection with pseudotyped HIV‐1_BaL_ virus

#### HIV‐1 BaL strain (HIV_BaL_) was used in some experiments

Vesicular stomatitis virus (VSV)‐G glycoprotein *in trans* with HIV‐1_BaL_ virion (VSVG‐HIV) were produced in 293T cells by cotransfection of VSVG expression plasmid and HIV_BaL_ plasmid in 293T cells using the calcium precipitation method. The HIV‐1_BaL_ plasmid and VSV‐G expression plasmid were obtained from the AIDS Research and Reference Reagent Program, Division of AIDS, NIAID, NIH. HFAs were infected with VSVG‐HIV at 10 ng or 20 ng of p24 overnight. Next day, cells were washed extensively with 1xDPBS and cultured in fresh astrocyte medium.

### Human fetal astrocytes (HFA) transfection of HIV‐1_BaL_ expression plasmid

Human fetal astrocytes (HFAs) were transiently transfected with pHIV‐1_BaL_ plasmid or control plasmid (pcDNA3) using Lipofectamine 2000 (Invitrogen). For experiments where HFAs were HIV‐infected and meth‐treated, meth was added to the cells during infection and thereafter at the specified concentrations.

### Gain and loss of β‐catenin function

To decrease β‐catenin levels in HFAs, siRNA specific to β‐catenin or a scrambled control siRNA was transfected in HFAs using Lipofectamine RNAiMAX reagent (Invitrogen) (Narasipura *et al*., 2012). To increase β‐catenin activities, a constitutively active β‐catenin mutant (S35Y), or a control plasmid (pcDAN3 + ) were transiently transfected using Lipofectamine 2000 reagent (Invitrogen). LiCl at 5 mm was used to augment β‐catenin activities as described (Schenkel *et al*., 2010).

### Senescence‐associated β‐galactosidase (SA‐βGal) cytochemical staining and quantitation

Senescence‐associated β‐galactosidase was detected by cytochemistry using SA‐βGal assay kit. Briefly, HFAs were washed with 1xDPBS, fixed with 1× fixation solution for 10‐15 min. After washing with 1xPBS twice, the cells were stained with X‐Gal containing staining solution at 37 °C overnight. Cells were then washed twice with 1xPBS, and covered with 70% glycerol. Cells were imaged using a Zeiss invert microscope with red, green, and blue channels under GRB setting. Acquired images were analyzed in ImageJ software. SA‐βGal^+^ cells as well as total cells were counted. A minimal of 200 cells were counted per treatment. SA‐βGal^+^ senescent cells were quantified as percentage of total cells.

### Western blotting analysis

HFAs and hippocampus tissues from human PBMC‐reconstituted NSG mice infected with HIV (see below) were lysed in 1xRIPA buffer in the presence of protease inhibitors (Sigma). 10 μg of cell lysates was loaded onto a 10% SDS‐PAGE for detection of β‐catenin and 20 μg lysates for p16^INK4A^ in 15% SDS‐PAGE. Proteins were separated in the gel by Tris–glycine system and transferred to a 0.45‐um nitrocellulose paper. The membrane was blocked with Pierce SuperBlock in PBS (Thermo Scientific, Waltham, MA) plus 0.15% Tween‐20 (Thermo Scientific) for 1 h at room temperature. Membranes were probed with primary antibodies with indicated dilutions in SuperBlock TW20 (0.12%). Primary antibodies used were as follows: rabbit anti‐p16^INK4A^ (1:500), mouse monoclonal anti‐active β‐catenin antibody (1:1000), rabbit anti‐β‐catenin antibody (1:50000), anti‐GFAP (1:2000), Iba1 (1:500), anti‐synaptophysin (1:2000), anti‐NF‐H (1:2000), anti‐GAPDH (1:50000). Membranes, after overnight incubation with indicated dilutions of primary antibodies in SuperBlock TW20 (0.12%), were washed three times for 45 min with TBS‐T and incubated with a 1:5000 dilution of anti‐mouse IgG or anti‐rabbit IgG‐HRP secondary antibody (Cell Signaling, Boston, MA) in SuperBlock T20 (0.1%) for 1 h at room temperature. Membranes were again washed three times for 45 min with TBS‐T and exposed to SuperSignal Femto ECL substrate reagent (Thermos Scientific) for p16^INK4A^ and active β‐catenin, and SuperSignal Pico ECL substrate reagent for total β‐catenin and GAPDH. Films were exposed and developed on a Konica SLX‐101A auto processor. Band densitometry was quantitated using ImageJ software (National Institutes of Health, Bethesda, MD), using GAPDH as loading controls.

### TOPflash assay

β‐catenin‐dependent transcription activities were analyzed using a T‐cell factor/lymphoid enhancer (TOPflash) reporter (Li *et al*., 2011). Transient transfections of the TOPflash reporter plasmids were performed using polyethylenimine reagent (Sigma). To circumvent the low infection rate of HIV in HFAs, HIV expression plasmid was cotransfected together with TOPflash plasmid to assess the effect of HIV on β‐catenin activities. Briefly, 1 μL of the transfection reagent was mixed with 100 μL of serum‐free media to which the recommended amount of DNA was added (0.5 μg per 100 000 cells). The resulting transfection mix was then incubated for 10 min at room temperature. Following the incubation, the transfection mix was added to the cells. 48 h after transfection, cells were lysed in 1× passive lysis buffer for 10 min. 20 μL of lysate was used in a luciferase reporter assay (Promega, Madison, WI) using a single injector luminometer. TOPflash activities were quantified based on luciferin arbitrary units per μg of total cell lysate.

### Cell cycle analysis

We adapted a cell cycle analysis using EdU Click‐It Plus Flow Cytometry kit (Invitrogen, CA) in combination with propidium iodide. Briefly, HFAs were treated with 10 μm of EdU for 16 h. HFAs were fixed in 1× Click‐It fixature solution for 15 min and permeabilized in the 1× permeabilization solution for 30 min in the dark. The Click‐It reaction with Alexa‐594 was performed in a 100‐μL reaction volume for 30 min. Subsequently, a solution of 40 ng mL^−1^ propidium iodide and 0.2 mg mL^−1^ RNase A was added to the cell pellet and the cells were kept at 4 °C overnight in the dark. Cells were analyzed in DB FASCCALIBUR, with Channel 594 for EdU. For the detection of PI with Alexa‐647, use 633/635‐nm excitation with a red emission filter (660/20 nm). Control cell samples for flow compensation analysis were without either EdU labeling, propidium iodide, or both. All data were analyzed in FlowJo software.

### Reconstitution of NSG mice with human PBMCs and infection with HIV_BaL_

These experiments were performed as described (Richards *et al*., 2016). Six‐ to eight‐week old NSG mice were injected with 2 × 10^7^ human PBMCs by intraperitoneal injection (i.p.). One to two weeks after reconstitution, mice were bled by retro‐orbital perfusion and extent of reconstitution was determined by flow cytometry. Reconstituted mice were infected with 10^4^ TCID_50_/mouse of HIV_BaL_ i.p.

### Isolation of astrocytes from mouse brain

Mice were anesthetized by inhalation of isoflurane and then perfused with 30–50 mL of ice‐cold PBS. Brains were collected and the hippocampus was lysed in 1xRIPA buffer for Western blotting analysis. Astrocytes were isolated as described (Lovatt *et al*., 2007). Briefly, brains were minced and digested with 8U mL^−1^ papain (Invitrogen, Carlsbad CA) and 80 kunitz units per mL DNase‐1 (Invitrogen) in Ca^2+^/Mg^2+^‐free PIPES cysteine buffer (Sigma) for 1 h at 37**°.** After 1 h, an additional 25 kunitz units per mL DNase‐1 was added for an additional 25 min. Digestion was then massed through a 70‐μm strainer with 1% BSA in DMEM and then overlayed onto a layer of isotonic 90% Percoll (Amersham, Piscataway, NJ) and spun at 200 g at 4 °C for 10 min. The myelin and Percoll layers were collected and myelin was removed using the Myelin Removal Bead Kit (Miltenyi Biotec, Cambridge, MA) according to the manufacturer's instructions.

### Flow cytometric analysis

Single cell suspensions of cells isolated from mouse brain were stained with LIVE/DEAD Fixable Violet Dead Cell Stain (Invitrogen) according to the manufacturer's instructions. Cells were washed with PBS and stained for CD45‐PE‐Cy7 and CD11b‐PE (BD Biosciences, San Jose, CA) for 30 min at 4**°** and then washed two times in PBS**.** Cells were fixed and permeabilized using BD Cytofix/Cytoperm (BD Biosciences) for 30 min at 4**°.** Cells were washed with Perm/Wash (BD Biosciences) according to the manufacturer's instructions. Cells were then stained with rat anti‐GFAP‐Alexa Fluor 488 and rabbit anti‐p16^INK4A^ (Proteintech, Chicago, IL) in BD Perm/Wash for 30 min at 4°. Cells were washed two times with BD Perm/Wash and then stained with an anti‐rabbit secondary antibody for the anti‐p16^INK4A^ conjugated to Alexa Fluor 647 for 30 min at 4 °C. Cells were washed two times with BD Perm/Wash and then one additional time with PBS before being run on a BD Fortessa Flow Cytometer (BD Biosciences). Astrocytes were identified as CD45^lo^CD11b^neg^GFAP^+^ and 2000 live astrocytes were collected. Flow cytometry was analyzed with FlowJo Analysis Software (Tree star, Inc., Ashland, OR).

### Meth treatment of rats

Adult male Sprague Dawley rats received meth (10 mg kg^−1^, i.p., every 2 h × 4) or saline control (1 mL kg^−1^, i.p., every 2 h × 4) as described (Halpin & Yamamoto, 2012). All rats were sacrificed 7 days after the treatment. This meth regimen was administrated based on previous studies to show that it caused long‐term neuronal damages similar to those observed in human meth abusers (Halpin & Yamamoto, 2012).

### SA‐βGal staining of rat brains

HIV‐1 Tg and control non‐Tg rats at 6 weeks of age, and rats of meth treatment were sacrificed, and brains were fixed in 4% paraformaldehyde overnight. Brain tissues were sectioned at 40 μm thickness. Brain sections were washed extensively in 1xPBS and then incubated in the sodium citrate buffer (pH 6.0) at 45 °C for 2 h. SA‐β‐Gal staining of these tissues was performed according to the manufacture's protocol (Cell Signaling).

### Statistical analysis

Comparisons between groups depending on experiment were analyzed using unpaired Student's t‐test, where appropriate. *P* values less than 0.05 were considered significant. All error bars are representative of standard deviation within the sample group. Experiments were repeated at least three times.

## Funding

This work was supported by R01 DA 033966 (LA), R01 NS060632 (LA), R01 NS084817 (XH), and DA007606 (BY). National Institutes of Health (Grant / Award Number: DA007606,R01 DA 033966,R01 NS060632 and R01 NS084817).

## Conflict of Interest

None declared.

## References

[acel12593-bib-0001] Acosta JC , Banito A , Wuestefeld T , Georgilis A , Janich P , Morton JP , Athineos D , Kang TW , Lasitschka F , Andrulis M , Pascual G , Morris KJ , Khan S , Jin H , Dharmalingam G , Snijders AP , Carroll T , Capper D , Pritchard C , Inman GJ , Longerich T , Sansom OJ , Benitah SA , Zender L , Gil J (2013) A complex secretory program orchestrated by the inflammasome controls paracrine senescence. Nat. Cell Biol. 15, 978–990. 23770676 10.1038/ncb2784 PMC3732483

[acel12593-bib-0002] Al‐Harthi L (2012) Wnt/beta‐catenin and its diverse physiological cell signaling pathways in neurodegenerative and neuropsychiatric disorders. J. Neuroimmune Pharmacol. 7, 725–730. 23114888 10.1007/s11481-012-9412-x PMC3690588

[acel12593-bib-0003] Baker DJ , Wijshake T , Tchkonia T , LeBrasseur NK , Childs BG , van de Sluis B , Kirkland JL , van Deursen JM (2011) Clearance of p16Ink4a‐positive senescent cells delays ageing‐associated disorders. Nature 479, 232–236. 22048312 10.1038/nature10600 PMC3468323

[acel12593-bib-0004] Benos DJ , Hahn BH , Shaw GM , Bubien JK , Benveniste EN (1994) gp120‐mediated alterations in astrocyte ion transport. Adv. Neuroimmunol. 4, 175–179. 7874384 10.1016/s0960-5428(06)80254-8

[acel12593-bib-0005] Berwick DC , Harvey K (2012) The importance of Wnt signalling for neurodegeneration in Parkinson's disease. Biochem. Soc. Trans. 40, 1123–1128. 22988876 10.1042/BST20120122

[acel12593-bib-0006] Bhat R , Crowe EP , Bitto A , Moh M , Katsetos CD , Garcia FU , Johnson FB , Trojanowski JQ , Sell C , Torres C (2012) Astrocyte senescence as a component of Alzheimer's disease. PLoS ONE 7, e45069. 22984612 10.1371/journal.pone.0045069 PMC3440417

[acel12593-bib-0007] Bishop CL , Bergin AM , Fessart D , Borgdorff V , Hatzimasoura E , Garbe JC , Stampfer MR , Koh J , Beach DH (2010) Primary cilium‐dependent and ‐independent Hedgehog signaling inhibits p16(INK4A). Mol. Cell 40, 533–547. 21095584 10.1016/j.molcel.2010.10.027

[acel12593-bib-0008] Campisi J (2013) Aging, cellular senescence, and cancer. Annu. Rev. Physiol. 75, 685–705. 23140366 10.1146/annurev-physiol-030212-183653 PMC4166529

[acel12593-bib-0009] Chana G , Everall IP , Crews L , Langford D , Adame A , Grant I , Cherner M , Lazzaretto D , Heaton R , Ellis R , Masliah E , Group H (2006) Cognitive deficits and degeneration of interneurons in HIV+ methamphetamine users. Neurology 67, 1486–1489. 17060582 10.1212/01.wnl.0000240066.02404.e6

[acel12593-bib-0010] Chinta SJ , Lieu CA , Demaria M , Laberge RM , Campisi J , Andersen JK (2013) Environmental stress, ageing and glial cell senescence: a novel mechanistic link to Parkinson's disease? J. Intern. Med. 273, 429–436. 23600398 10.1111/joim.12029 PMC3633085

[acel12593-bib-0011] Deeks SG , Verdin E , McCune JM (2012) Immunosenescence and HIV. Curr. Opin. Immunol. 24, 501–506. 22658763 10.1016/j.coi.2012.05.004

[acel12593-bib-0012] Delmas V , Beermann F , Martinozzi S , Carreira S , Ackermann J , Kumasaka M , Denat L , Goodall J , Luciani F , Viros A , Demirkan N , Bastian BC , Goding CR , Larue L (2007) Beta‐catenin induces immortalization of melanocytes by suppressing p16INK4a expression and cooperates with N‐Ras in melanoma development. Genes Dev. 21, 2923–2935. 18006687 10.1101/gad.450107 PMC2049194

[acel12593-bib-0013] Elzi DJ , Song M , Hakala K , Weintraub ST , Shiio Y (2012) Wnt antagonist SFRP1 functions as a secreted mediator of senescence. Mol. Cell. Biol. 32, 4388–4399. 22927647 10.1128/MCB.06023-11 PMC3486147

[acel12593-bib-0014] Florian MC , Nattamai KJ , Dorr K , Marka G , Uberle B , Vas V , Eckl C , Andra I , Schiemann M , Oostendorp RA , Scharffetter‐Kochanek K , Kestler HA , Zheng Y , Geiger H (2013) A canonical to non‐canonical Wnt signalling switch in haematopoietic stem‐cell ageing. Nature 503, 392–396. 24141946 10.1038/nature12631 PMC4078992

[acel12593-bib-0015] Halpin LE , Yamamoto BK (2012) Peripheral ammonia as a mediator of methamphetamine neurotoxicity. J. Neurosci. 32, 13155–13163. 22993432 10.1523/JNEUROSCI.2530-12.2012 PMC3464918

[acel12593-bib-0016] Henderson LJ , Sharma A , Monaco MC , Major EO , Al‐Harthi L (2012) Human immunodeficiency virus type 1 (HIV‐1) transactivator of transcription through its intact core and cysteine‐rich domains inhibits Wnt/beta‐catenin signaling in astrocytes: relevance to HIV neuropathogenesis. J. Neurosci. 32, 16306–16313. 23152614 10.1523/JNEUROSCI.3145-12.2012 PMC3508723

[acel12593-bib-0017] Hser YI , Huang D , Brecht ML , Li L , Evans E (2008) Contrasting trajectories of heroin, cocaine, and methamphetamine use. J. Addict. Dis. 27, 13–21. 10.1080/10550880802122554 PMC2821675 18956525

[acel12593-bib-0018] Inestrosa NC , Montecinos‐Oliva C , Fuenzalida M (2012) Wnt signaling: role in Alzheimer disease and schizophrenia. J. Neuroimmune Pharmacol. 7, 788–807. 23160851 10.1007/s11481-012-9417-5

[acel12593-bib-0019] Iudicello JE , Woods SP , Vigil O , Scott JC , Cherner M , Heaton RK , Atkinson JH , Grant I , Group HIVNRC (2010) Longer term improvement in neurocognitive functioning and affective distress among methamphetamine users who achieve stable abstinence. J. Clin. Exp. Neuropsychol. 32, 704–718. 20198527 10.1080/13803390903512637 PMC2911490

[acel12593-bib-0020] Jackson AR , Shah A , Kumar A (2014) Methamphetamine alters the normal progression by inducing cell cycle arrest in astrocytes. PLoS ONE 9, e109603. 25290377 10.1371/journal.pone.0109603 PMC4188627

[acel12593-bib-0021] Kang C , Xu Q , Martin TD , Li MZ , Demaria M , Aron L , Lu T , Yankner BA , Campisi J , Elledge SJ (2015) The DNA damage response induces inflammation and senescence by inhibiting autophagy of GATA4. Science 349, aaa5612. 26404840 10.1126/science.aaa5612 PMC4942138

[acel12593-bib-0022] Kennedy BK , Berger SL , Brunet A , Campisi J , Cuervo AM , Epel ES , Franceschi C , Lithgow GJ , Morimoto RI , Pessin JE , Rando TA , Richardson A , Schadt EE , Wyss‐Coray T , Sierra F (2014) Geroscience: linking aging to chronic disease. Cell 159, 709–713. 25417146 10.1016/j.cell.2014.10.039 PMC4852871

[acel12593-bib-0023] Langford D , Adame A , Grigorian A , Grant I , McCutchan JA , Ellis RJ , Marcotte TD , Masliah E , Group HIVNRC (2003) Patterns of selective neuronal damage in methamphetamine‐user AIDS patients. J. Acquir. Immune Defic. Syndr. 34, 467–474. 14657756 10.1097/00126334-200312150-00004

[acel12593-bib-0024] Levchenko A , Davtian S , Freylichman O , Zagrivnaya M , Kostareva A , Malashichev Y (2015) Beta‐catenin in schizophrenia: possibly deleterious novel mutation. Psychiatry Res. 228, 843–848. 26027441 10.1016/j.psychres.2015.05.014

[acel12593-bib-0025] Li W , Henderson LJ , Major EO , Al‐Harthi L (2011) IFN‐gamma mediates enhancement of HIV replication in astrocytes by inducing an antagonist of the beta‐catenin pathway (DKK1) in a STAT 3‐dependent manner. J. Immunol. 186, 6771–6778. 21562161 10.4049/jimmunol.1100099 PMC3167069

[acel12593-bib-0026] Lovatt D , Sonnewald U , Waagepetersen HS , Schousboe A , He W , Lin JH , Han X , Takano T , Wang S , Sim FJ , Goldman SA , Nedergaard M (2007) The transcriptome and metabolic gene signature of protoplasmic astrocytes in the adult murine cortex. J. Neurosci. 27, 12255–12266. 17989291 10.1523/JNEUROSCI.3404-07.2007 PMC6673251

[acel12593-bib-0027] Minagar A , Shapshak P , Fujimura R , Ownby R , Heyes M , Eisdorfer C (2002) The role of macrophage/microglia and astrocytes in the pathogenesis of three neurologic disorders: HIV‐associated dementia, Alzheimer disease, and multiple sclerosis. J. Neurol. Sci. 202, 13–23. 12220687 10.1016/s0022-510x(02)00207-1

[acel12593-bib-0028] Narasipura SD , Henderson LJ , Fu SW , Chen L , Kashanchi F , Al‐Harthi L (2012) Role of beta‐catenin and TCF/LEF family members in transcriptional activity of HIV in astrocytes. J. Virol. 86, 1911–1921. 22156527 10.1128/JVI.06266-11 PMC3302377

[acel12593-bib-0029] Nath A , Hauser KF , Wojna V , Booze RM , Maragos W , Prendergast M , Cass W , Turchan JT (2002) Molecular basis for interactions of HIV and drugs of abuse. J. Acquir. Immune Defic. Syndr. 31(Suppl. 2), S62–S69. 12394784 10.1097/00126334-200210012-00006

[acel12593-bib-0030] Pu C , Vorhees CV (1993) Developmental dissociation of methamphetamine‐induced depletion of dopaminergic terminals and astrocyte reaction in rat striatum. Brain Res. Dev. Brain Res. 72, 325–328. 8097974 10.1016/0165-3806(93)90201-k

[acel12593-bib-0031] Purohit V , Rapaka R , Shurtleff D (2011) Drugs of abuse, dopamine, and HIV‐associated neurocognitive disorders/HIV‐associated dementia. Mol. Neurobiol. 44, 102–110. 21717292 10.1007/s12035-011-8195-z

[acel12593-bib-0032] Reid W , Sadowska M , Denaro F , Rao S , Foulke J Jr , Hayes N , Jones O , Doodnauth D , Davis H , Sill A , O'Driscoll P , Huso D , Fouts T , Lewis G , Hill M , Kamin‐Lewis R , Wei C , Ray P , Gallo RC , Reitz M , Bryant J (2001) An HIV‐1 transgenic rat that develops HIV‐related pathology and immunologic dysfunction. Proc. Natl Acad. Sci. USA 98, 9271–9276. 11481487 10.1073/pnas.161290298 PMC55410

[acel12593-bib-0033] Richards MH , Narasipura SD , Kim S , Seaton MS , Lutgen V , Al‐Harthi L (2015) Dynamic interaction between astrocytes and infiltrating PBMCs in context of neuroAIDS. Glia 63, 441–451. 25331637 10.1002/glia.22763 PMC4293283

[acel12593-bib-0034] Richards MH , Narasipura SD , Seaton MS , Lutgen V , Al‐Harthi L (2016) Migration of CD8 + T Cells into the central nervous system gives rise to highly potent anti‐HIV CD4dimCD8bright T Cells in a Wnt signaling‐dependent manner. J. Immunol. 196, 317–327. 26582945 10.4049/jimmunol.1501394 PMC4685022

[acel12593-bib-0035] Saylor D , Dickens AM , Sacktor N , Haughey N , Slusher B , Pletnikov M , Mankowski JL , Brown A , Volsky DJ , McArthur JC (2016) HIV‐associated neurocognitive disorder ‐ pathogenesis and prospects for treatment. Nat. Rev. Neurol. 12, 309. 27080521 10.1038/nrneurol.2016.53 PMC5842923

[acel12593-bib-0036] Schenkel JM , Zloza A , Li W , Narasipura SD , Al‐Harthi L (2010) Beta‐catenin signaling mediates CD4 expression on mature CD8 + T cells. J. Immunol. 185, 2013–2019. 20631314 10.4049/jimmunol.0902572 PMC3963465

[acel12593-bib-0037] Scholz D , Poltl D , Genewsky A , Weng M , Waldmann T , Schildknecht S , Leist M (2011) Rapid, complete and large‐scale generation of post‐mitotic neurons from the human LUHMES cell line. J. Neurochem. 119, 957–971. 21434924 10.1111/j.1471-4159.2011.07255.x

[acel12593-bib-0038] Scott JC , Woods SP , Matt GE , Meyer RA , Heaton RK , Atkinson JH , Grant I (2007) Neurocognitive effects of methamphetamine: a critical review and meta‐analysis. Neuropsychol. Rev. 17, 275–297. 17694436 10.1007/s11065-007-9031-0

[acel12593-bib-0039] Shah A , Silverstein PS , Kumar S , Singh DP , Kumar A (2012) Synergistic cooperation between methamphetamine and HIV‐1 gsp120 through the P13K/Akt pathway induces IL‐6 but not IL‐8 expression in astrocytes. PLoS ONE 7, e52060. 23251686 10.1371/journal.pone.0052060 PMC3522628

[acel12593-bib-0040] Sharma A , Hu XT , Napier TC , Al‐Harthi L (2011) Methamphetamine and HIV‐1 Tat down regulate beta‐catenin signaling: implications for methampetamine abuse and HIV‐1 co‐morbidity. J. Neuroimmune Pharmacol. 6, 597–607. 21744004 10.1007/s11481-011-9295-2 PMC3714216

[acel12593-bib-0041] Talloczy Z , Martinez J , Joset D , Ray Y , Gacser A , Toussi S , Mizushima N , Nosanchuk JD , Goldstein H , Loike J , Sulzer D , Santambrogio L (2008) Methamphetamine inhibits antigen processing, presentation, and phagocytosis. PLoS Pathog. 4, e28. 18282092 10.1371/journal.ppat.0040028 PMC2242831

[acel12593-bib-0042] Wang Z , Pekarskaya O , Bencheikh M , Chao W , Gelbard HA , Ghorpade A , Rothstein JD , Volsky DJ (2003) Reduced expression of glutamate transporter EAAT2 and impaired glutamate transport in human primary astrocytes exposed to HIV‐1 or gp120. Virology 312, 60–73. 12890621 10.1016/s0042-6822(03)00181-8

[acel12593-bib-0043] Ye X , Zerlanko B , Kennedy A , Banumathy G , Zhang R , Adams PD (2007) Downregulation of Wnt signaling is a trigger for formation of facultative heterochromatin and onset of cell senescence in primary human cells. Mol. Cell 27, 183–196. 17643369 10.1016/j.molcel.2007.05.034 PMC2698096

